# Rethinking the extrinsic incubation period of malaria parasites

**DOI:** 10.1186/s13071-018-2761-4

**Published:** 2018-03-12

**Authors:** Johanna R. Ohm, Francesco Baldini, Priscille Barreaux, Thierry Lefevre, Penelope A. Lynch, Eunho Suh, Shelley A. Whitehead, Matthew B. Thomas

**Affiliations:** 1 Center for Infectious Disease Dynamics, Pennsylvania State University, University Park, PA USA; 2 Institute of Biodiversity Animal Health and Comparative Medicine, University of Glasgow, Glasgow, Scotland UK; 3 MIVEGEC, IRD, CNRS, University of Montpellier, Montpellier, France; 4 College of Life and Environmental Sciences, Penryn Campus, University of Exeter, Cornwall, UK

**Keywords:** Malaria, Mosquito, Extrinsic incubation period, EIP, Temperature

## Abstract

The time it takes for malaria parasites to develop within a mosquito, and become transmissible, is known as the extrinsic incubation period, or EIP. EIP is a key parameter influencing transmission intensity as it combines with mosquito mortality rate and competence to determine the number of mosquitoes that ultimately become infectious. In spite of its epidemiological significance, data on EIP are scant. Current approaches to estimate EIP are largely based on temperature-dependent models developed from data collected on parasite development within a single mosquito species in the 1930s. These models assume that the only factor affecting EIP is mean environmental temperature. Here, we review evidence to suggest that in addition to mean temperature, EIP is likely influenced by genetic diversity of the vector, diversity of the parasite, and variation in a range of biotic and abiotic factors that affect mosquito condition. We further demonstrate that the classic approach of measuring EIP as the time at which mosquitoes first become infectious likely misrepresents EIP for a mosquito population. We argue for a better understanding of EIP to improve models of transmission, refine predictions of the possible impacts of climate change, and determine the potential evolutionary responses of malaria parasites to current and future mosquito control tools.

**Electronic supplementary material:**

The online version of this article (10.1186/s13071-018-2761-4) contains supplementary material, which is available to authorized users.

## Background

The extrinsic incubation period (EIP) of malaria, also called the period of sporogony, describes the time it takes for parasites to develop in the mosquito from point of ingestion *via* an infected blood meal, through to the point at which sporozoites enter the salivary glands and the mosquito becomes infectious. In the classic models of malaria transmission (e.g. [1–4]), the EIP is one of the most influential parameters because it interacts with adult mosquito survival rate as an exponential term, meaning that even very small changes in EIP can have a large effect on the number of mosquitoes living long enough to be able to transmit parasites. Changes in EIP potentially have much greater impact than equivalent changes in traits such as vector competence (i.e. the ability of vectors to become infectious) or vector density. Despite its epidemiological importance, EIP remains poorly characterized.

Our current understanding of EIP derives largely from research conducted in the early to mid-1900s, wherein the development times of the human malaria parasites *Plasmodium falciparum*, *P. vivax* and *P. malariae* were examined in the mosquito *Anopheles maculipennis* across a range of constant temperatures [5]. These data were used to construct degree-day models to predict the EIP of malaria parasites at a given environmental temperature [1, 2]. The models assume it takes a set number of accumulated degree days (DD) for malaria parasites to complete their development once mean daily temperature (T, in degrees Celsius) exceeds a lower temperature threshold for development (T_min_). For *P. falciparum*, DD = 111 and T_min_ = 16 °C, giving EIP = 111/(T-16) [1]. For *P. vivax* the equivalent is EIP = 105/(T-14.5), and for *P. malariae* EIP = 144/(T-16) [1].

The Detinova degree-day models of *P. falciparum* and *P. vivax* described above have become lore [1]. Many contemporary studies that provide an estimate of EIP do so without acknowledging the source, let alone attempting any direct validation. However, in spite of their widespread use, the assumptions underpinning these models have received little attention. For instance, Nikolaev [5] defined EIP as the time at which sporozoites were first observed in the salivary glands of an individual infected mosquito, yet whether this is the most relevant measure in terms of overall transmission potential of a mosquito population is not clear. The degree-day models also assume that EIP can be estimated using mean temperatures alone. Whether other factors, such as parasite and vector genetics, or other sources of environmental variation, also play a role has been virtually ignored. Equally, whether there is a genetic basis for variation in EIP and potential for evolution in parasite development rate under different environmental conditions (e.g. in response to vector control interventions or climate change) is unknown. Our aim in the current paper is to examine these assumptions in order to improve our understanding of EIP, identify key knowledge gaps, and motivate further work to better characterize EIP moving forward.

## What factors determine the EIP?

### The influence of temperature

The original work of Nikolaev evaluated EIP of different malaria species across a range of constant temperatures (from 19–20 °C to 30 °C for *P. falciparum*, and 15–16 °C to 30 °C for *P. vivax*) [5]. In nature, however, mosquitoes do not live at a constant temperature but experience daily temperature fluctuations. There is now significant literature indicating that daily temperature variation can have a substantial effect on mosquito and parasite life history, beyond the effects of mean temperature [6–13]. In particular, theory and empirical evidence indicate that daily temperature fluctuations are likely to have the greatest influence toward the upper and lower thermal limits, with daily variation acting to increase parasite development rate under low mean temperatures, slowing development rate under high mean temperatures, and potentially having no net effect under intermediate conditions [6, 14, 15]. Thus, estimates of EIP derived under constant temperatures may not reflect the actual EIPs occurring in nature.

We are aware of no explicit empirical tests of how daily temperature variation impacts EIP of human malaria parasites. However, studies using a rodent malaria confirm the contrasting effects of daily temperature variation on parasite development rate under cool and warm conditions [6], so it seems likely that EIP of human malaria parasites could be similarly affected. Studies on dengue virus development within *Aedes aegypti* also show that temperature fluctuations shorten EIP under cool mean temperatures, but that fluctuations have no effect on virus development rate at higher temperatures, despite reduced vector competence [13].

**Fig. 1:**
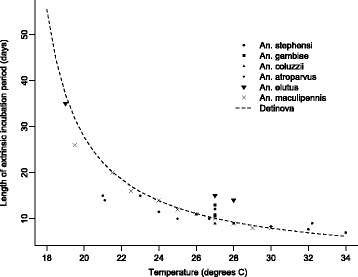
Empirical estimates of EIP for *P. falciparum* across a range of studies. The dotted black line represents the standard degree-day model of Detinova [1] parameterized using the data for *An. maculipennis* [5]. Data points of the same shape indicate the same mosquito species but may derive from more than one study. The data are extracted from Mordecai et al. [21] (and references therein [26, 56]), together with Shapiro et al. [23], Nikolaev [5], Hien et al. [57] and Kligler & Mer [58]. Note that different studies vary in methods for estimating EIP. Though most report EIP as the time until first observation of sporozoites following an infectious feed, data points from [23] are derived from median EIP

Current degree-day models also define a lower temperature threshold below which development ceases. For *P. falciparum* T_min_ = 16 °C. However, the lowest temperature measured for *P. falciparum* in the studies of Nikolaev was 19–20 °C [5]*,* and T_min_ = 16 °C was selected by Detinova [1] based on the earlier work of Moshkovsky [2] who fitted a linear regression to the parasite development rate data of Nikolaev and found the line crossed the x-axis at 16 °C. Other studies provide varied estimates of the lower thermal thresholds of human malaria, ranging from as low as 15 °C to as high as 24–26 °C for *P. falciparum*, and from 14.5 °C to 17.5 °C for *P. vivax* (see [16] and Table 1.3 in [17]). Few studies attempt to estimate EIP of *P. falciparum* at temperatures below 20 °C (Fig. 1). Thus, whether we can define 16 °C as the appropriate lower developmental threshold as used in the Detinova model [1] is currently unknown. This lack of knowledge is striking given that T_min_ is so integral to the degree-day model approach.

Additionally, the degree-day models assume the relationship between parasite development rate (the reciprocal of the EIP) and temperature is linear [1, 2]. In contrast, a number of recent theoretical studies describe malaria parasite development rate as a unimodal, non-linear function [18–21]. Which of these approaches is most appropriate depends critically on whether there is an optimum temperature for development and whether the rate declines as temperatures increase above this optimum. Unfortunately, current evidence is again limited. The studies adopting unimodal functions include data from a very limited number of historic studies where dissection of mosquitoes at high temperatures revealed no sporozoites in the salivary glands (e.g. [22]). However, the absence of sporozoites does not necessarily equate to zero growth rate. This distinction is not simply semantic; we ought to know whether high temperatures limit transmission because of a decline in vector competence (which could be direct parasite mortality or perhaps mediated *via* the mosquito), or because parasite growth slows and EIP becomes progressively longer. In the recent study of Shapiro et al. [23] there was no evidence of a non-linear decline in parasite development rate up to 34 °C, even though the proportion of mosquitoes becoming infectious declined at temperatures above 27 °C. More data are needed to resolve this fundamental issue.

### Parasite genetic diversity

Nikolaev’s study [5] identified differences in the EIP between three *Plasmodium* species. Other studies have further demonstrated interspecific variation in EIP [24–26]. The genetic basis for these differences in EIP is poorly understood. Additionally, whether there is intraspecific variation in EIP between parasite genotypes is unknown. The Detinova degree-day models assume no intraspecific variation but we are not aware of any empirical studies investigating this assumption.

Studies from other vector-borne pathogens provide some evidence of intraspecific variation in EIP. For example, the emergence of a new dominant genotype of West Nile virus in North America has been attributed to the new genotype having a shorter EIP in *Culex* mosquito vectors compared to the original strain [27]. Similarly, the Southeast Asian genotype of dengue serotype 2 virus has displaced the American genotype in several countries [28] which has been explained by its shorter EIP resulting in an estimated 2- to 65-fold increase in the vectorial capacity of the *Ae. aegypti* vector [29]. Additionally, differences in dissemination rate of three strains of dengue serotype 2 viruses within the same *Ae. aegypti* colony have also been observed [30], further suggesting that the pathogen’s intraspecific variation in EIP is genetically influenced. However, intraspecific variation in EIP is not always observed among viruses. A single mutation between two isolates of chikungunya virus (CHIKV) favored transmissibility by *Aedes albopictus* [31] and has been associated with an outbreak that occurred in Indian Ocean territories, but no quantitative differences in EIPs between these strains were observed [32]. In addition, a statistical analysis aimed at estimating the relationship between temperature and EIP in three orbiviruses transmitted by *Culicoides* biting midges showed that the rate of virus replication was mostly consistent among the different pathogen genotypes [33].

Given the high levels of genetic variation within malaria parasite species [34–38], it seems likely that there could be genotypic variation for EIP [39]. Different *Plasmodium* genotypes have been shown to vary in their capacity to infect a specific mosquito species [40, 41], possibly due to different immune evasion mechanisms [42]. Additionally, parasite growth rates within the vertebrate host are under genetic control [39, 42]. Better characterizing intraspecific variation in sporogony could improve investigation of local transmission dynamics (e.g. [43]) and could help in understanding the spread of drug resistant genotypes (cf. [44, 45]).

### Vector genetic diversity

There are approximately 70 species of mosquitoes in the genus *Anopheles* known to contribute to transmission of malaria parasites to humans [46]. The current degree-day models of EIP were derived from studies on one population of a single species, the Eurasian vector *Anopheles maculipennis* [5]*.* Few researchers would be happy to accept that all populations or species of *Anopheles* mosquitoes are equally permissive to malaria infection, and there has been substantial research investment to understand the genetic mechanisms underlying variation in susceptibility/refractoriness (e.g. [36, 47, 48]). Yet for EIP the prevailing assumption is that all vector species and populations are identical and the EIP is a property of the parasite response to mean temperature alone. Indeed, White & Rao [49] state “for lack of any evidence to the contrary, it must be assumed that differences in vector species does [*sic*] not affect the results [of EIP]”.

In Fig. 1 we present all the available data we can find from studies that have explicitly measured EIP of *P. falciparum* (note that we followed the approach of Mordecai et al. [21] and excluded studies if they did not demonstrate adequate control of temperature, were unclear on parasite species, or had insufficient sample size such as reporting infections from dissection of single mosquitoes). The figure reveals that data are extremely sparse and that certain empirical estimates of EIP do not clearly match the standard degree-day model. Whether there are significant differences between vector species is impossible to say as there are insufficient data to generate species-specific EIP models for any of the key malaria vectors in Africa, Asia or Latin America.

In addition to the potential for interspecific differences in EIP between vectors (Fig. 1), there is the potential for intraspecific variation. In a recent study, Ye et al. [50] examined EIP of dengue across 40 genetically distinct families of *Aedes aegypti*. They showed significant differences in EIP (measured as time to detectable virus in the saliva) between families ranging from 4–14 days, and that variation in EIP was highly heritable (~40%). Shorter EIPs were additionally correlated with shorter vector lifespans and higher virulence. This work demonstrates that EIP of dengue is largely controlled by variation in the mosquito genome. We are aware of no studies on malaria vectors examining intraspecific genetic variation in EIP. The data from Shapiro et al. [23] indicate differences between individual mosquitoes but the mechanisms are unclear. However, with evidence for genetic influence on other aspects of malaria parasite infection such as resistance/susceptibility [35, 47, 48, 51], interactions with insecticide resistance [52], and vector genotype × parasite genotype interactions [40, 53, 54], it would be surprising if there was no influence of mosquito genetics on EIP.

### Other biotic and abiotic factors

The complex interplay between parasite and vector traits that determine overall transmission can be influenced by many factors [55–58]. Larval food limitation has been shown to decrease malaria parasite survival [59] and affect infection prevalence and intensity [60, 61]. The mechanisms behind these observations are not well understood but could be linked to altered immune response, resource allocation within the vector [59–61], or effects on adult body size that influence the blood meal volume and hence the number of infecting parasites (note that temperatures in the larval environment also impact ultimate adult size [62]). Importantly, quality of the larval habitat has been shown to affect EIP for both dengue [63] and *P. falciparum* [23] independent of temperature.

Food intake by adult mosquitoes can also affect parasite development. Relatively few studies have looked at the impact of sugar feeding on mosquito or parasite life history but there is evidence that nectar from different plants can potentially inhibit or enhance parasite load and rate of parasite development [57, 64]. Blood-feeding has also recently been shown to influence EIP of dengue virus in *Aedes* mosquitoes, with additional blood meals accelerating virus development [65].

Malaria parasites potentially compete with many organisms inside mosquitoes [66–69], including mixed infections with other malaria parasite genotypes [70, 71]. These interactions can impact parasite establishment and density *via* competition or immune-mediate mechanisms [72]. What effect they might have on EIP of malaria parasites is not known, but for dengue, the presence of an intracellular bacterial parasite (*Wolbachia*) has been shown to extend the EIP [73, 74]. There is further potential for parasite/pathogen-mediated effects *via* trans-generational immune priming, which can confer lasting protection within an individual [75] and in its offspring [76]. If parental exposure to parasites has consequences for malaria parasite resistance in the offspring [76], it is possible this could impact EIP, though this has not yet been explored.

### How should EIP be measured?

The original work of Nikolaev [5] dissected mosquitoes at various time points following an infectious blood meal and defined EIP as the time at which sporozoites were first observed in mosquito salivary glands. Capturing the time of the first mosquitoes to become infectious might make sense: mosquitoes that allow rapid parasite migration through their bodies have more opportunities to infect humans at subsequent bites and so might be the most epidemiological relevant individuals. Equally, if parasite development is highly synchronized between individuals, then the time to first infection will likely be a reasonable approximation for the mosquito population as a whole. On the other hand, if development is widely distributed between mosquitoes, then a few early infectious mosquitoes might be unrepresentative of the total mosquito population, and be a poor predictor for force of infection.

A number of recent empirical studies (e.g. [23, 57]) have demonstrated that the time parasites take to reach the salivary glands is not the same for all mosquitoes in a population, even if they have received the same infectious blood meal and are maintained under identical conditions, again highlighting the need to understand sources of this variation [54]. Furthermore, variance in EIP and the median EIP value are affected by temperature [23]. Under warm conditions the median is shorter and there is less variation in duration of sporogony between mosquitoes, but as conditions cool, the median increases and the time between the first and last mosquitoes to become infectious can extend to several days, widening the distribution of EIP [23].

**Fig. 2:**
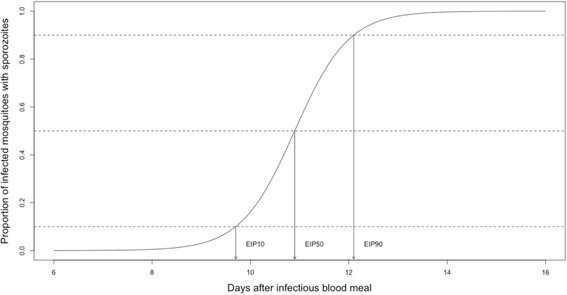
Proportion of malaria-infected mosquitoes with sporozoites present in the salivary glands (i.e. becoming infectious) over time following an infectious blood meal. Here the dynamics of EIP are characterized using a logistic model following the approach of Paaijmans et al. [77] and Shapiro et al. [23, 60] (and see also data in Hien et al. [57]). The conventional way of estimating EIP is to measure the time at which sporozoites first appear in salivary glands of infected mosquitoes (approximating the EIP_10_). However, given EIP is not perfectly synchronized between individual mosquitoes, the EIP could equally be characterized using alternative measures such as the median value for the mosquito population (EIP_50_), or the time at which the maximum proportion of the population become infectious (approximating the EIP_90_). In this illustrative example we assume all infected mosquitoes go on to become infectious. If conversion efficiency of oocysts to sporozoites is less than 100%, the asymptote will be reduced

In Additional file 1, we present an outline of a model developed to examine whether different measures of EIP affect estimates of the probability that mosquitoes live long enough to become infectious. We based our analysis on the study of Shapiro et al. [23], which measured the EIP of *P. falciparum* in *An. stephensi* across six constant temperatures ranging from 21–34 °C. Briefly, the dynamics of sporogony were characterized by a logistic function (Fig. 2, Additional file 1), which enables us either to define individual measures of EIP (the 10-percentile, 50-percentile or 90-percentile), or to represent the full growth kinetics of parasites across the mosquito population.

**Fig. 3:**
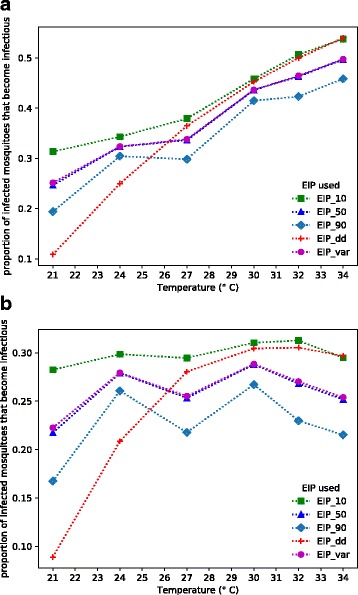
The proportion of infected mosquitoes predicted to survive the duration of EIP and be able to transmit *P. falciparum* parasites at different temperatures. The EIP_var_ values refer to the full logistic models describing the dynamics of sporogony across six constant temperatures presented in Shapiro et al. [23]. The EIP_10_, EIP_50_ and EIP_90_ values represent the 10-, 50- and 90-percentile points from the logistic curves. The EIP_dd_ values are from the classic Detinova degree-day model [1]. **a** Assumes a constant mortality rate of adult mosquitoes of 10% per day. **b** Assumes adult mortality rate to vary with temperature based on the data presented in Shapiro et al. [23]

In order to examine the proportion of infected mosquitoes that survive through the different measures of EIP we needed to estimate adult mosquito mortality rate. Many transmission models assume a constant daily mortality rate. In Fig. 3a we weight the proportion of mosquitoes that developed sporozoites at each temperature by the proportion that survived through sporogony for a constant mortality rate of 10% per day, comparing the EIP_10_, EIP_50_, EIP_90_, the standard degree-day model, and the full logistic model (Additional file 1: Table S1). In Fig. 3b we conduct a similar analysis but rather than assume a constant daily mortality rate we used the actual temperature-dependent mortality rates measured by Shapiro et al. [23] for each of the six temperatures (Additional file 1: Table S2).

Comparison of Fig. 3a with 3b shows that the pattern of adult mortality has a qualitative effect on the proportion of infected mosquitoes predicted to be alive and infectious for our different measures of EIP. With constant daily mortality rate there is a general trend for the proportion of infectious mosquitoes to increase as temperatures rise, since warmer temperatures shorten EIP whichever way it is characterized. With temperature-dependent mortality, however, the proportion of infectious mosquitoes tends to fall as temperature extends beyond 27 °C, since reductions in EIP are offset by increases in daily mosquito mortality rates at higher temperatures.

In addition, regardless of how mortality is estimated, at low temperatures the standard degree-day model tends to underestimate the probability of mosquitoes being alive and infectious compared to the estimates based on the empirical data of Shapiro et al. [23]. This difference largely derives from the fact that Shapiro et al. [23] reported more rapid sporogony than Nikolaev [5] at cooler temperatures. At temperatures above 27 °C the degree-day model increasingly approximates the EIP_10_, which is to be expected as Nikolaev [5] estimated EIP from the first few mosquitoes to become infectious (which is close to the EIP_10_) and the data of Shapiro et al. [23] and Nikolaev [5] are more similar at high temperatures. Perhaps most important is that the EIP_50_ yields almost identical values to approximations based on the full logistic model, while the EIP_10_ and EIP_90_ tend to over and under estimate the probability of a mosquito being alive and infectious, respectively. This result indicates that it is important to characterize the full dynamics of sporogony and that the distribution of EIP is better estimated using the median EIP (EIP_50_), rather than beginning or end points of the distribution. This is not how EIP has been interpreted for almost a century.

## Conclusions

Current understanding of EIP of malaria parasites is limited. There are very few empirical data and those that exist tend to report EIP inappropriately. Moreover, basic information regarding the genetic and environmental determinants of EIP is lacking. This is unfortunate as the potential environmental and genetic influences are numerous and likely to have profound evolutionary and epidemiological implications [77–83]. One obvious implication is that the intensity of malaria transmission will vary spatially and temporally depending on environmental fluctuations and specific vector-parasite combinations. It could be that effect sizes are small and that the established degree-day models capture the variation in EIP across time and space adequately. However, it could also be that mosquito species, mosquito condition, parasite strain, etc. have a substantial influence. This should not be an open question. There has been considerable speculation regarding possible impacts of climate warming on malaria transmission [19, 21, 81, 83], yet the effects could depend as much on the specifics of the local mosquito-parasite pairing as the absolute change in temperature itself. More empirical studies are required to rigorously examine EIP both as a stand-alone trait, and in the context of other essential components of vectorial capacity, such as mosquito density, adult longevity, and biting rate, which all contribute to overall transmission. Such studies would be facilitated greatly by the optimization of non-destructive methodologies allowing fine temporal resolution of EIP within individual mosquitoes, as is now possible for arboviruses [50, 84, 85]. In terms of transmission dynamics, it would also be valuable to determine the parasite’s ability to adjust its development rate in response to environmental cues (adaptive phenotypic plasticity). For instance, can malaria parasites adaptively speed up their EIP when their transmission is compromised by the imminent death of their vectors (perhaps in old mosquitoes, those exposed to insecticides, or in the presence of competing parasites)? In a related way, given transmission is ultimately dependent on the bite of an infectious mosquito, it would be interesting to explore whether EIP could potentially be linked to biting rate and gonotrophic cycle. Like EIP, biting behavior is influenced by a suite of environmental factors [86, 87] and it is possible that the duration of EIP is rhythmically modulated to avoid the situation where the parasite is ready to be transmitted but the mosquito is not ready to feed, either because the mosquito is in the middle of a gonotrophic cycle [7] or because it is physiologically constrained [87, 88]. Such condition-dependent developmental strategies have been described in blood-stage malaria parasites [89, 90] and deserve considerations in infected mosquitoes. Finally, understanding the extent to which EIP is genetically variable is also crucial to understanding the capacity of EIP to evolve in response to malaria interventions or mosquito life history, as genetic variation fuels evolution. Current core vector control tools (long-lasting insecticide-treated bed nets (LLINs) and indoor residual insecticide sprays (IRS)) act, in part, by changing mosquito population age structure [91, 92]. These tools exploit the fact that the EIP is long relative to the lifespan of most mosquitoes, and that mosquitoes take multiple blood meals throughout their lifetime. By increasing the probability of mortality per blood feeding event, LLINs and IRS reduce the number of mosquitoes that live long enough for the parasite to complete EIP. Other prospective control tools also target the ‘old infectious’ mosquitoes [91, 92]. There is now a substantial industry built around understanding and managing the evolutionary responses of mosquitoes to insecticides and other vector control tools (e.g. see [93]). Whether vector control tools can drive evolutionary changes in EIP and select for parasite clones with shorter EIPs is unknown but should, perhaps, become part of an extended insecticide-resistance monitoring process. The fitness of parasites should increase with shorter EIP, unless faster developing parasites inflict higher mortality costs on mosquitoes or come with fitness trade-offs to the parasite such as reduced infectivity (as discussed in [54]). Whether mosquito fitness is affected by EIP length of malaria parasites is unknown. The implications for transmission could depend on relationships with relevant transmission traits such as mosquito longevity or parasite load [54]. For example, are fast developing clones also those that are the most virulent and reduce mosquito longevity? Are fast developing parasites also those that produce the fewest transmissible stages? These potential trade-offs and constraints may have important implications for understanding the evolutionary potential of EIP. More broadly, the effects of parasite drug resistance and mosquito insecticide resistance - two important sources of genetic variation - on EIP deserve attention.

## Additional file

Additional file 1: **Text.** Numerical approximation of proportion of vectors surviving to become infectious assuming logistic EIP. **Table S1.** Comparison of results for approximation of probability of surviving from infection to infectiousness using logistic model. Results used for plots in main text are highlighted. Chosen *D*_*max*_ and *δ* give results consistent to 6dp with results from ten times smaller *δ* and *D*_*max*_ of 100 *vs* 30, indicating that for the intended purpose, no material benefit would be gained from using smaller *δ* or larger *D*_*max*._ **Table S2.** The temperature-related values used for *k, tM*, and *μ* , taken from Shapiro et al [23]. (DOC 68 kb)

## Abbreviations

EIP: Extrinsic incubation period
EIP_10_: Extrinsic incubation period measured as the time until 10 percent of infected mosquitoes become infectious
EIP_50_: Extrinsic incubation period measured as the time until 50 percent of infected mosquitoes become infectious which equates to the median time of sporozoite development
EIP_90_: Extrinsic incubation period measured as the time until 90 percent of infected mosquitoes become infectious
