# Supplementary material for: Rethinking the extrinsic incubation period of malaria parasites
Source: Parasit Vectors. 2018 Mar 12;11:178. doi: 10.1186/s13071-018-2761-4 (PMC5848458; doi:10.1186/s13071-018-2761-4)
Supplement: Supplementary file 1 — Text. Numerical approximation of proportion of vectors surviving to become infectious assuming logistic EIP. Table S1. Comparison of results for approximation of probability of surviving from infection to infectiousness using logistic model. Results used for plots in main text are highlighted. Chosen Dmax and δ give results consistent to 6dp with results from ten times smaller δ and Dmax of 100 vs 30, indicating that for the intended purpose, no material benefit would be gained from using smaller δ or larger Dmax. Table S2. The temperature-related values used for k, tM, and μ , taken from Shapiro et al [23]. (DOC 68 kb) [file 13071_2018_2761_MOESM1_ESM.doc]

# Additional file 1

# Numerical approximation of proportion of vectors surviving to become infectious assuming logistic EIP

The model we used to determine the proportion of infected mosquitoes surviving to become infectious is based on different measures of EIP and adult mosquito mortality rate.

To predict the number of infected mosquitoes surviving long enough to become infectious after an infectious blood meal, we constructed a model based on the logistic models fitted by Shapiro et al. [1] for the observed cumulative proportion of the vector population infectious *t* days after an infectious feed. Briefly, the proportion of infected mosquitoes which are infectious (have sporozoites in salivary glands) by time *t*, is predicted by the logistic relationship between the rate at which they become infectious (*k*) and time described in Equation 1.

**Equation 1**

Proportion of infected individuals which are infectious by time *t* = $\frac{1}{1+e^{-k\left(t-t_{50}\right)}}$

Here, *t* = time in days after the initial infectious blood feed, *k* = rate constant, and *t*_50_ = equivalently the time at which 0.5*b* of the population has reached infectiousness, or the time of maximal increase in proportion infectious.

Logistic functions of EIP for 6 temperatures were taken from the empirical data presented in Shapiro et al. [1] (note this study presented data for 2 experimental blocks and we base our analysis on data from block 1, although the results are qualitatively equivalent for block 2).

Unlike a single value for EIP, the use of a distribution for the time to EIP precludes generation of a meaningful value without making an assumption regarding vector mortality rates because mortality disproportionately affects the later maturing infections. Combining the logistic model with survival functions is not analytically tractable, but it is possible to numerically generate values for the proportion of infected vectors surviving to reach infectiousness at a given temperature with given mortality assumptions, using Equation 2.

Equation 2

Proportion of infected vectors which survive to become infectious =

$$\sum_{n=0}^{\frac{D_{\max}}{\delta}} e^{-\mu n\delta}\left(\frac{1}{1+e^{-k\left(\left(n+1\right)\delta-t_{M}\right)}}-\left(\begin{matrix} \text{if }n\text{ =0 0} \\ \text{if }n\text{ >0 }\frac{1}{1+e^{-k\left(n\delta-t_{M}\right)}} \end{matrix}\right)\right)$$

Here *D*_max_ is a number of days greater than the maximum number required to reach the maximum proportion of infectious mosquitoes following an infectious blood feed. *δ* is a time period of less than one day, short enough to give results to a sufficient level of accuracy. Other variables are the same as for Equation 1.

To examine the interaction between different measures of EIP and mosquito mortality on the proportion of vectors surviving to become infectious, we assumed either an instantaneous daily mortality rate of 10%, or we used the actual temperature-dependent mortality rates from the study of Shapiro et al. that provided the data for the logistic models [1].

# Additional details on the numerical approximation of proportion of vectors surviving to become infectious assuming logistic EIP

*t* represents time since infectious blood meal

$b_{\max}$represents the total proportion of vectors infected by blood meal (ie proportion which will become infectious if they survive long enough to do so).

$t_{M}=t_{50}$ , the time at which 50% of infected vectors have become infectious

*k* = rate for fitted logistic models

Based on the fitted logistic models the cumulative probability an exposed vector is infectious at time *t* =$\frac{b_{\max}}{1+e^{-k\left(t-t_{M}\right)}}$

The cumulative probability an infected vector is infectious at time *t* therefore =$\frac{1}{1+e^{-k\left(t-t_{M}\right)}}$

Cumulative probability infected vector becomes infectious between times *t*_1_ and *t*_2_ (if it survives to do so) is therefore

= $\frac{1}{1+e^{-k\left(t_{2}-t_{M}\right)}}-\frac{1}{1+e^{-k\left(t_{1}-t_{M}\right)}}$

Cumulative probability that a vector is alive at time *t* is $e^{-\mu t}$

To approximate the proportion of infected vectors which survive and transition to infectiousness, we calculate the probability of becoming infectious during a given time period (*δ*), multiplied by the probability of surviving to that time period. We consider survival to the start rather than end of the relevant time period, since this is more conservative, giving an overestimate rather than underestimate of the number of vectors which achieve infectiousness.

This gives;

$$\sum_{n=0}^{\frac{D_{\max}}{\delta}} e^{-\mu n\delta}\left(\frac{1}{1+e^{-k\left(\left(n+1\right)\delta-t_{M}\right)}}-\left(\begin{matrix} \text{if }n\text{ =0 0} \\ \text{if }n\text{ >0 }\frac{1}{1+e^{-k\left(n\delta-t_{M}\right)}} \end{matrix}\right)\right)$$

The different treatment at *n*=0 is to avoid omitting a small fraction of infectious vectors as a result of the non-zero value generated by the logistic function at *t*=0.

Values are chosen for *δ*, the interval over which each proportion reaching infectiousness is calculated, and $D_{\max}$, the highest value of *t* included in the calculation, which give an acceptable level of accuracy in the results. The values used for figures in the main text were generated using $D_{\max}$=30 days and *δ* = 1 second. These values were tested with the data and found to give a level of accuracy sufficient for the plots produced, as illustrated in Table S1.

Table S1 Comparison of results for approximation of probability of surviving from infection to infectiousness using logistic model. Results used for plots in main text are highlighted. Chosen *D_max_* and *δ* give results consistent to 6dp with results from ten times smaller *δ*  and *D_max_* of 100 vs 30, indicating that for the intended purpose, no material benefit would be gained from using smaller *δ* or larger *D_max_*

| *using Shapiro et al B1* | | | Temperature | | | | | |
| --- | --- | --- | --- | --- | --- | --- | --- | --- |
| mortality | *D_max_*  /days | *δ* | 21 | 24 | 27 | 30 | 32 | 34 |
| 0.1 | 30 | 0.1 second | 0.251455 | 0.323463 | 0.337874 | 0.436361 | 0.464372 | 0.497614 |
| 0.1 | 30 | 1 second | 0.251455 | 0.323463 | 0.337874 | 0.436361 | 0.464372 | 0.497614 |
| 0.1 | 100 | 1 second | 0.251455 | 0.323463 | 0.337874 | 0.436361 | 0.464372 | 0.497614 |
|  |  |  |  |  |  |  |  |  |

**Table S2** The temperature-related values used for *k, t_M_*, and *μ* , taken from Shapiro et al

|  | 21°C | 24°C | 27°C | 30°C | 32°C | 34°C |
| --- | --- | --- | --- | --- | --- | --- |
| *k* | 0.92 | 3.52 | 1.83 | 4.8 | 2.37 | 2.82 |
| *t_M_* | 14.0 | 11.3 | 10.9 | 8.3 | 7.7 | 7.0 |
| *μ* | 0.109 | 0.113 | 0.126 | 0.15 | 0.171 | 0.197 |

1. Shapiro LLM, Whitehead SA, Thomas MB. Quantifying the effects of temperature on mosquito and parasite traits that determine the transmission potential of human malaria. PLoS Bio. 2017;15(10): e2003489
